# Detection of *Bartonella henselae* DNA in the blood of patients with livedoid vasculopathy^⋆^

**DOI:** 10.1016/j.abd.2022.07.007

**Published:** 2023-03-25

**Authors:** Marina Rovani Drummond, Luciene Silva dos Santos, Lais Bomediano Souza, Gabriela Nero Mitsuushi, Maria Letícia Cintra, Andrea Fernandes Eloy da Costa França, Elemir Macedo de Souza, Paulo Eduardo Neves Ferreira Velho

**Affiliations:** a Department of Clinical Medicine, School of Medical Sciences, Universidade Estadual de Campinas, Campinas, São Paulo, SP, Brazil; b Laboratory of Applied Research in Dermatology and Bartonella Infection, School of Medical Sciences, Universidade Estadual de Campinas, Campinas, São Paulo, SP, Brazil; c Department of Medicine, Pontifícia Universidade Católica de Campinas, Campinas, São Paulo, SP, Brazil; d Department of Pathological Anatomy, School of Medical Sciences, Universidade Estadual de Campinas, Campinas, São Paulo, SP, Brazil

**Keywords:** Bartonella, Livedoid vasculopathy, Skin ulcer

## Abstract

**Background:**

Livedoid vasculopathy (LV) manifests as ulcers and atrophic white scars on the lower extremities. The main known etiopathogenesis is hypercoagulability with thrombus formation, followed by inflammation. Thrombophilia, collagen and myeloproliferative diseases may induce LV, but the idiopathic (primary) form predominates. *Bartonella* spp. may cause intra-endothelial infection and skin manifestations caused by these bacteria may be diverse, including leukocytoclastic vasculitis and ulcers.

**Objective:**

The aim of this study was to investigate the presence of bacteremia by *Bartonella* spp. in patients with difficult-to-control chronic ulcers diagnosed as primary LV.

**Methods:**

Questionnaires and molecular tests (conventional PCR, nested PCR and real-time PCR) were applied and liquid and solid cultures were performed in the blood samples and blood clot of 16 LV patients and 32 healthy volunteers.

**Results:**

*Bartonella henselae* DNA was detected in 25% of LV patients and in 12.5% of control subjects but failed to reach statistically significant differences (*p* = 0.413).

**Study limitations:**

Due to the rarity of primary LV, the number of patients studied was small and there was greater exposure of the control group to risk factors for *Bartonella* spp. infection.

**Conclusion:**

Although there was no statistically significant difference between the groups, the DNA of *B. henselae* was detected in one of every four patients, which reinforces the need to investigate *Bartonella* spp. in patients with primary LV.

## Introduction

Livedoid vasculopathy (LV) manifests as painful ulcers located distally on the lower limbs, which slowly develop into whitish atrophic lesions, with punctate telangiectasias, brownish pigmentation, accompanied by livedo racemosa. The most often accepted LV etiopathogenesis comprises vaso-occlusive phenomena resulting from hypercoagulable states. Conditions such as thrombophilia, diffuse connective tissue diseases , myeloproliferative disorders, and blood stasis are known to be associated with the disease, but the idiopathic (primary) form is the predominant one.^1^, ^2^

**Figure 1:**
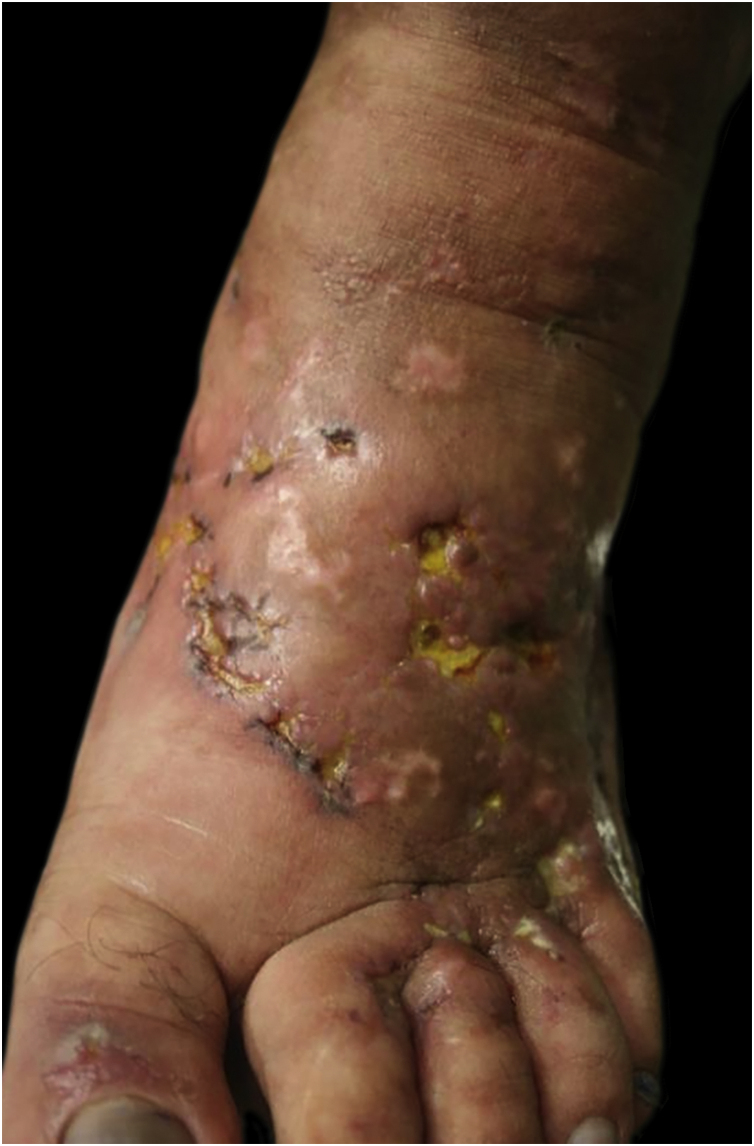
Right foot, aspect of the dorsum one year after onset of the condition: mild livedo racemosa, hypochromic atrophic scars with telangiectasias, ulcers with erythematous background, some covered by fibrinopurulent tissue and others by hematic crusts.

**Figure 2:**
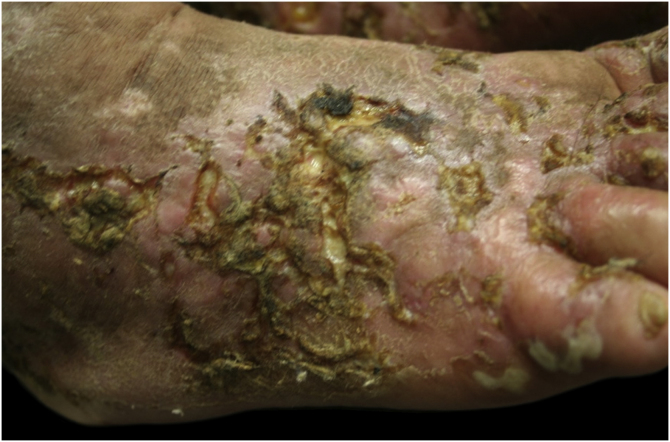
Right foot, aspect of the dorsum: pustules and ulcers with erythematous background, many crusts and concretions, hypochromic atrophic lesions and other hyperchromic ones.

**Figure 3:**
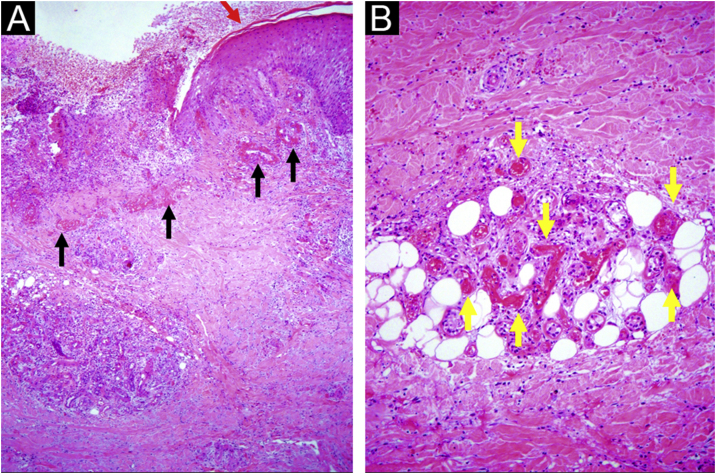
Chronic ulcer: (A) hyperplastic epidermis at the edge (red arrow), vessels with hyalinized walls at the edge and in the ulcer bed (black arrows), inflammatory cell aggregates and fibrosis of the reticular dermis and hypodermis; (B) wall hyalinosis and occlusive thrombosis of the lumen in the dermis and hypodermis vessels (yellow arrows), with inflammatory exudate containing polymorphonuclear neutrophils. Hematoxylin & eosin, x40 (A) and x100 (B).

This study was carried out based on the clinical observation of a 41-year-old patient, with a history of contact with cats and who had ulcerated lesions on the lower limbs that were difficult to control for 12 years (Figure 1, Figure 2). Histopathology showed hypodermitis associated with leukocytoclastic vasculitis and the presence of frequent hyaline thrombi, with fibrinoid necrosis of small vessel walls. Mucin and hemosiderin deposits were also observed, in addition to vascular proliferation (Fig. 3). After excluding other diseases, the patient was managed as a case of primary LV and treated over the years with vasodilators, antiplatelet agents, corticosteroids, and cycles of antibiotics (metronidazole, penicillin, and ciprofloxacin), with transient improvement. Considering the strong epidemiology of contact with cats, screening for *Bartonella* spp. was suggested. *Bartonella henselae* DNA was detected by polymerase chain reaction (PCR) in the patient's blood and there was a prompt improvement of the chronic ulcers with treatment of the *B. henselae* infection with doxycycline 200 mg/day for one year. After treatment withdrawal, however, the LV lesions recurred. The bacterium was isolated and its DNA was once again detected in a new blood sample (Table 1).

**Table 1:** Results of molecular investigations in the index case of primary livedoid vasculopathy with bacteremia confirmed by *Bartonella henselae.*

Sample	Whole blood		Liquid culture		Solid culture	
	Conventional PCR (ITS)	Nested PCR (*fstZ*)	Conventional PCR (ITS)	Nested PCR (*fstZ*)	Conventional PCR (ITS)	Nested PCR (*fstZ*)
November/2012 (under antibiotic treatment)	**+**	*−*	*−*	*−*	**No growth**	**No growth**
December/2013 (without antibiotic treatment)	**+**	*−*	**+**	**+**	**+**	**+**

+, detected DNA; *−*, DNA not detected.

*Bartonella* spp. are fastidious bacteria and, therefore, difficult to isolate. Although distributed worldwide, they are neglected and life-threatening microorganisms that often live inside erythrocytes and endothelial cells. Infection by these bacteria can be asymptomatic, but can also cause a variety of clinical conditions in humans, including fever of unknown origin, endocarditis, and cat-scratch disease.^3^ Cutaneous manifestations also vary and include leukocytoclastic vasculitis and ulcers, as reviewed by Lins et al. in a recent review of the cutaneous manifestations of bartonellosis published in this journal.^4^ The main objective of the present study was to investigate the presence of *Bartonella* spp. in patients with primary LV with difficult-to-control ulcers.

## Methods

A free and informed consent form was obtained from each participant after evaluation and authorization provided by the UNICAMP Institutional Research Board (CAAE: 48163015.8.0000.5405). Patients over 18 years of age, with a diagnosis of primary LV undergoing follow-up at UNICAMP Dermatology Outpatient Clinic were included in the study, and volunteers without clinical complaints were included as a control group (UNICAMP students and/or employees, over 18 years of age and not pregnant). Each participant answered a questionnaire to assess risk exposure for acquiring *Bartonella* spp. infection. Blood samples were collected from the patients and control individuals using an aseptic technique in tubes containing ethylenediaminetetraacetic acid (EDTA) and in tubes without anticoagulant.

**Figure 4:**
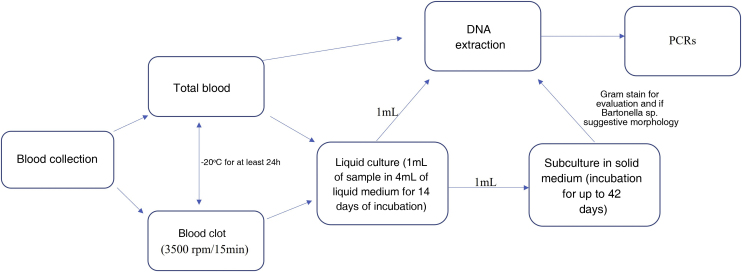
Flowchart of the procedures performed.

The laboratory where the analyses were performed follows standards to ensure the quality control described by Pitassi et al.^5^ Liquid cultures of blood and blood clot samples (blood clots collected from the tube without anticoagulant) and solid cultures of all liquid cultures were performed as previously described.^6^, ^7^ DNA was extracted from blood samples, from blood clots, and from the liquid cultures using QIAamp DNA minikit (Qiagen Inc., USA). In addition to conventional PCR for the constitutive mammalian gene, GAPDH, genus-specific conventional PCR for *Bartonella* spp. and species-specific double-amplification (nested) and real-time PCRs for *B. henselae* were performed. Three controls were added in all procedures, (extraction negative control, PCR-negative control and PCR-positive control ‒ strain 3715 of *B. henselae* registered in the Culture Collection Section of Instituto Adolfo Lutz). Additionally, dilutions of *B. henselae* were tested in each reaction to determine the sensitivity and the limit of detection of each PCR. All of these methods have been previously described.^6^, ^7^, ^8^, ^9^, ^10^, ^11^ The primers used in the molecular investigation of patients and volunteers are described in Table 2. The obtained amplifications that showed adequate quality for sequencing were sent for similarity comparison with previously deposited material. The flowchart of the performed procedures is shown in Fig. 4.

**Table 2:** Primers used in the molecular investigation of the patient and control groups.

PCR	Primer	Sequence of nucleotides (5'-3')	Molecular target
Conventional^9^	GAPDH F	CCTTCATTGACCTCAACTACAT	GAPDH
	GAPDH R	CCAAACTTGTCATGGATGACC	
Conventional^10^	ITS F	CTTCAGATGATGATCCCAAGCCTTYTGGCG	ITS
	ITS R	GAACCGACGACCCCCTGCTTGCAAAGCA	
Double amplification (nested)^11^	BH F	GCCGCAAAGTTCTTTTCATG	*ftsZ*
	BH R	AGGTGAACGCGCTTGTATTTG	
	BH S	CAAAACGGTTGGAGAGCGT	
	BH A	CGCCTGTCATCTCATCAAGA	
Real-time^8^	CS F	ATGGGTTTTGGTCATCGAGT	*gltA*
	CS R	AAATCGACATTAGGGTAAAGTTTTT

## Results

Sixteen patients with primary LV and 32 individuals without clinical complaints participated in the study. All answered a questionnaire whose data are shown in Table 3. It can be observed that the control group was younger (*p* = 0.001), with a mean age of 26.71 years, whereas the mean age was 37.62 years among the patients. The control group had more men than the patient group (*p* = 0.048). Individuals in this group had more contact with pets (*p* = 0.007), were more bitten and/or scratched (*p* = 0.000), and were more exposed to arthropods (*p* = 0.003). There was no significant difference (*p* > 0.05) when comparing these characteristics in those who had *Bartonella* spp. DNA detected and those who did not, in each of the groups separately.

**Table 3:** Comparison between the control group and the group of patients with livedoid vasculopathy.

Variable	Control (n = 32)	Livedoid vasculopathy (n = 16)	Total (n = 48)	*p*-value
Age (Mean ± SD)	26.71 ± 8.57	37.62 ± 9.76	30.35 ± 10.29	**0.001**^a^
Age (Median (min‒max)	23.00 (18.0–51.0)	39.50 (15.0–51.0)	28.50 (15.0–51.0)	
Sex				
Female	19 (39.6%)	14 (29.2%)	33 (68.8%)	**0.048**^c^
Male	13 (27.1%	2 (4.2%)	15 (31.2%)	
Total	32 (66.7%)	16 (33.3%)		
Contact with housepets				
Yes	5 (10.6%)	9 (19.1%)	14 (29.8%)	**0.007**^b^
No	26 (55.3%	7 (14.9%)	33 (70.2%)	
Total	31 (66.0%)	16 (34.0%)		
Contact with wild animals				
Yes	4 (8.3%)	0 (0.0%)	4 (8.3%)	0.286^b^
No	28 (58.3%)	16 (33.3%)	44 (91.7%)	
Total	32 (66.7%)	16 (33.3%)		
Bite or Scratch				
Yes	24 (50.0%)	3 (6.2%)	27 (56.2%)	**0.000**^b^
No	7 (14.6%)	13 (27.1%)	20 (41.7%)	
Missing	1 (2.1%)	0 (0.0%)	1 (2.1%)	
Total	32 (66.7%)	16 (33.3%)		
Exposure to arthropods				
Yes	29 (60.4%)	8 (16.7%)	37 (77.1%)	**0.003**^b^
No	3 (6.2%)	8 (16.7%)	11 (22.9%)	
Total	32 (66.7%)	16 (33.3%)		
Detection of *Bartonella henselae* DNA				
Yes	4 (8.35%)	4 (8.3%)	8 (16.7%)	0.413^b^
No	28 (58.3%)	12 (25.0%)	40 (83.3%)	
Total	32 (66.7%)	16 (33.3%)		

*p*-values shown in **bold** in the tables are statistically significant.

a Mann-Whitney test.

b Fisher’s test.

c Chi-square test.

The constitutive gene (GAPDH) was detected in all samples from patients and volunteers, guaranteeing the quality of the DNA extracted for the analyses and the absence of inhibitors for the reactions, guaranteeing the quality of the DNA extracted from the samples and the absence of inhibitors for the molecular reactions.

In conventional PCR for *Bartonella* spp.*,* the limit of detection was 50 genome equivalent (GE) and ten GE in the nested PCR and real-time PCR.

*B. henselae* DNA was detected in four patients in at least one species-specific reaction. In the control group, DNA was detected in four volunteers. Amplified material from six participants out of eight with bacterial DNA detection was sequenced, and all amplified samples showed 99% to 100% of similarity to *B. henselae* (GenBank accession number: CP020742.1).

There was no growth of *Bartonella* spp. in solid cultures from any sample of the 48 participants. There was no statistical significance using Fischer’s test in the detection of bacterial DNA between the two groups (*p* = 0.413), as also observed in Table 3.

Considering that primary LV is a rare manifestation and using Fisher's exact test to establish the sample size calculation for the study and setting the significance level at 5% (alpha or type I error) with a sampling power of 80% (beta or type II error of 20%), based on the obtained data, it can be established that the minimum sample size for the results to have statistical significance would be 106 patients.

## Discussion

The authors did not find any studies in the literature reporting infection by *Bartonella* spp. in LV. As it has been proposed for SARS-CoV-2,^12^ it is possible that persistent infection by *Bartonella* spp. also causes direct vascular damage or promotes a hypercoagulable state.

It is known that LV is a vaso-occlusive disease with hyalinization and thrombosis of dermal vessels, and many patients complain of pain and paresthesia. All studied patients reported severe pain associated with the presence of ulcers, and most reported paresthesia or mild pain after healing.^1^ Several articles have associated chronic pain with *Bartonella* spp. infection.^13^, ^14^, ^15^ Translational studies have documented pain hypersensitivity in mice infected with *B. henselae*.^16^, ^17^ These microorganisms cause intra-endothelial infection and could stimulate endothelin (ET-1) synthesis, a potent vasoconstrictor, which is related to paresthesia and pain, including chronic pain.^13^, ^16^, ^18^, ^19^

It was not possible to detect the DNA of *Bartonella* spp. in three-quarters of the patients, which is not surprising, since LV is a cutaneous manifestation of different diseases. Moreover, *B. henselae* causes cyclic bacteremia, and the blood samples can be obtained at intervals when the bacteria are not circulating. Different authors have shown that infection by *Bartonella* spp. has a greater chance of being diagnosed through a biopsy of tissue fragments than by a blood sample and that the results of this difference may be of greater importance and show statistical significance.^19^, ^20^, ^21^, ^22^, ^23^ For ethical reasons, only blood samples were collected to minimize damage to the control group volunteers.

Furthermore, there is no reference diagnostic method for *Bartonella* spp. infection and false-negative results are frequent, even when using a platform with molecular and microbiological techniques. The need for multiple tests for adequate detection of this microorganism, aiming at reducing false negative results, has already been discussed by Drummond et al. Molecular techniques are limited by their low sensitivity (minimum of 4,000 copies/mL in the laboratory where the analyses were performed).^6^ Even in diseases with tissue involvement, the identification of the agent in the anatomopathological examination has low sensitivity.^4^ Historically, silver impregnation stains have been used for the detection of *Bartonella* species; however, as the stain is non-specific, the reading can be difficult due to the formation of silver precipitates. A study by Caponetti et al. evaluated the use of immunohistochemistry (IHC) for the identification of *B. henselae* in cat-scratch disease (CSD) in 24 cases of lymphadenitis in formalin-fixed and paraffin-embedded biopsy specimens and compared the results with silver impregnation (SI) and PCR. The positive cases were as follows: SI, 11 (46%); PCR, nine (38%); and IHC, six (25%). Only two cases (8%) were positive for all three studies and SI was more sensitive but less specific. Although 11 (46%) of the study cases were positive using SI, only six of these samples were concurrently positive at the immunohistochemical analysis and/or PCR. This finding suggests that several of the cases interpreted as positive by SI may represent false-positive results and that the diagnostic sensitivity of these three tests is low for CSD.^24^ Another study with 106 cases of endocarditis compared the results of immunohistochemistry, Western blotting and real-time PCR in whole blood, serum and valvular tissue. Immunohistochemistry and Warthin-Starry staining were the least sensitive techniques. In this study, Warthin-Starry staining was the more sensitive of these two methods (five samples were positive for Warthin-Starry staining and negative in the immunohistochemical analysis). However, this histological stain is not specific for *Bartonella* spp. As the infectious process can be localized, a negative immunohistochemical result does not definitively rule out the diagnosis of endocarditis by *Bartonella* spp.^20^ The isolation of *Bartonella* spp. is difficult, even under specific and ideal conditions.

Bacteremia was documented in the index case, through the isolation of the bacteria in a solid medium even after one year of treatment with doxycycline, but no isolation of *Bartonella* spp. was observed in any of the study participants. The difficulty of treating the infection with antibiotics, as seen in trench fever (caused by *Bartonella quintana*) and Oroya fever (which is the first manifestation of biphasic Carrion's disease and can progress to *verruga Peruana*, caused by *Bartonella bacilliformis*), suggest that these bacteria are not eradicated. This potential persistence of the infection makes it impossible to guarantee that manifestations observed in LV are infectious or post-infectious immune reactions, as has been suggested.^25^

Few research groups perform solid cultures for the isolation of *Bartonella* spp. This is due to the fact that isolation depends on specific media and prolonged incubation. Additionally, fastidious growth and lack of success in primary isolation make this type of diagnosis unfeasible.

As LV is the cutaneous manifestation of different diseases, infection by *Bartonella* spp. could be just one of the causes of this manifestation. Moreover, because it infects endothelial cells and is the only known genus of bacteria to stimulate its proliferation in humans,^26^ the screening in blood samples may not represent the patient's actual condition in relation to the infection by these bacteria, as bacteremia is usually cyclical.

Data collected from the questionnaire regarding the risk factors for bartonellosis (Table 3) showed that the volunteers in the control group were significantly younger since most of these volunteers consisted of UNICAMP students. LV affects more women and there was a statistical difference between the sex proportion in the groups. One limitation of the study is the fact that the volunteers were more exposed to risk factors for acquiring *Bartonella* spp. infection, factors that were observed in a study carried out with blood donors from the UNICAMP Blood Bank,^27^ since they reported, in greater numbers, when compared to the patients, having suffered bites and scratches, contact with pets and exposure to arthropods. Even with lower risk exposure, the group of patients with LV had twice the infection rate, 25% (4/16), which may indicate that the prevalence of infection by *B. henselae* in patients is higher and that these bacteria can, in fact, be related to disease onset. Another explanation for not having found a statistically significant difference is the small number of patients in the study that evaluates patients with a rare disease, which was carried out as a pilot study in a single institution. As described in the results, using volunteers with the same characteristics as the controls, would take almost seven times more patients to document statistical significance.

The lack of a reference diagnostic test, combined with the great difficulty in detecting bacteremia caused by *Bartonella* spp., reinforces the need to use diverse and complementary methods to increase the sensitivity and accuracy of the diagnosis. This combination of methods and the testing of different samples makes the laboratory diagnosis more effective and has been used by different research groups.^6^, ^20^, ^28^, ^29^, ^30^

## Conclusion

*B. henselae* DNA was detected in one out of four patients with primary LV, which reinforces the need to investigate *Bartonella* spp. infection in these individuals, although there was no statistical difference between the patient group and the control group.

## Financial support

National Council for Scientific and Technological Development (CNPq) for the scholarships granted: n. 170501/2018-3 (LSS), n. 313762/2021-0 (PENFV) and n. 151006/2021-0 (MRD). São Paulo Dermatology Support Fund (Funadersp) / Brazilian Society of Dermatology, Regional São Paulo (França, AFEC).

## Authors' contributions

Marina Rovani Drummond: Approval of the final version of the manuscript; drafting and editing of the manuscript; critical review of the literature; critical review of the manuscript.

Luciene Silva dos Santos: Approval of the final version of the manuscript; design and planning of the study; drafting and editing of the manuscript; critical review of the literature; critical review of the manuscript.

Lais Bomediano Souza: Approval of the final version of the manuscript; drafting and editing of the manuscript; critical review of the manuscript.

Gabriela Nero Mitsuushi: Approval of the final version of the manuscript; design and planning of the study; critical review of the literature; participation in patient selection and sample collection.

Maria Letícia Cintra: Approval of the final version of the manuscript; drafting and editing of the manuscript; critical review of the literature; critical review of the manuscript.

Andrea Fernandes Eloy da Costa França: Approval of the final version of the manuscript; drafting and editing of the manuscript; critical review of the literature; critical review of the manuscript.

Elemir Macedo de Souza: Approval of the final version of the manuscript; drafting and editing of the manuscript; critical review of the literature; critical review of the manuscript.

Paulo Eduardo Neves Ferreira Velho: Approval of the final version of the manuscript; design and planning of the study; participation in patient selection and sample collection; drafting and editing of the manuscript; critical review of the literature; critical review of the manuscript.

## Conflicts of interest

None declared.
